# Anti-MDA5 Antibody Linking COVID-19, Type I Interferon, and Autoimmunity: A Case Report and Systematic Literature Review

**DOI:** 10.3389/fimmu.2022.937667

**Published:** 2022-06-27

**Authors:** Antonio Tonutti, Francesca Motta, Angela Ceribelli, Natasa Isailovic, Carlo Selmi, Maria De Santis

**Affiliations:** ^1^ Department of Biomedical Sciences, Humanitas University, Pieve Emanuele, Italy; ^2^ Istituto di Ricerca e Cura a Carattere Scientifico (IRCCS) Humanitas Research Hospital, Rozzano, Italy; ^3^ Division of Rheumatology and Clinical Immunology, Istituto di Ricerca e Cura a Carattere Scientifico (IRCCS) Humanitas Research Hospital, Rozzano, Italy

**Keywords:** COVID-19, type I interferon signature, anti-MDA5 syndrome, inflammatory myositis, immunology, autoimmune disease, cytokines

## Abstract

**Introduction:**

The SARS-CoV-2 infection has been advocated as an environmental trigger for autoimmune diseases, and a paradigmatic example comes from similarities between COVID-19 and the myositis-spectrum disease associated with antibodies against the melanoma differentiation antigen 5 (MDA5) in terms of clinical features, lung involvement, and immune mechanisms, particularly type I interferons (IFN).

**Case Report:**

We report a case of anti-MDA5 syndrome with skin manifestations, constitutional symptoms, and cardiomyopathy following a proven SARS-CoV-2 infection.

**Systematic Literature Review:**

We systematically searched for publications on inflammatory myositis associated with COVID-19. We describe the main clinical, immunological, and demographic features, focusing our attention on the anti-MDA5 syndrome.

**Discussion:**

MDA5 is a pattern recognition receptor essential in the immune response against viruses and this may contribute to explain the production of anti-MDA5 antibodies in some SARS-CoV-2 infected patients. The activation of MDA5 induces the synthesis of type I IFN with an antiviral role, inversely correlated with COVID-19 severity. Conversely, elevated type I IFN levels correlate with disease activity in anti-MDA5 syndrome. While recognizing this ia broad area of uncertainty, we speculate that the strong type I IFN response observed in patients with anti-MDA5 syndrome, might harbor protective effects against viral infections, including COVID-19.

## Introduction

Infections are well known triggers for autoimmune diseases through different proposed mechanisms, including bystander activation, cross-reactivity, epitope spreading, and cryptic antigen unmasking (1–3).

New-onset autoimmune diseases following COVID-19 have been described, including both single-organ (e.g. Guillain-Barré syndrome) (4) and systemic rheumatologic diseases (e.g. inflammatory arthritis, connective tissue diseases, and vasculitis) (5). Anti-melanoma differentiation antigen 5 (anti-MDA5) syndrome is a disease belonging to the spectrum of inflammatory myositis (6); this is a heterogeneous group of systemic autoimmune disorders, characterized by skeletal muscle inflammation (7). Historically, myositis has been classified according to Bohan and Peter’s criteria (8). A currently accepted classification divides myositis into clinical-serological categories: dermatomyositis, antisynthetase syndrome, immune-mediated necrotizing myopathies, polymyositis, and inclusion-body myopathy. Concerning muscle pathology, dermatomyositis is characterized by B and CD4 T cell infiltrate with perivascular distribution and complement activation, whereas in polymyositis inflammation appears at endomysial level, with a mononuclear cell infiltrate mainly composed by CD8 T cells and macrophages (7, 9–11). However, since each category might include heterogenous entities, a classification system based on myositis-specific antibodies has been advocated (6). Anti-MDA5 syndrome has usually been classified as dermatomyositis, due to prominent skin manifestations, mild (seldom absent) muscle involvement (clinically amyopathic dermatomyositis), and interstitial pneumonitis (6, 7, 12). The clinical picture of anti-MDA5 syndrome is unique, with digital ulcers, palmar and plantar papules, signs of vasculitis, and severe pulmonary involvement, associated with anti-MDA5 autoantibodies (12). A clinical overlap between COVID-19 and anti-MDA5 syndrome has been described, especially in the terms of rapidly progressive interstitial lung disease (ILD), fever, myalgia, and skin rashes; also, imaging findings show significant similarities, i.e. bilateral ground-glass pneumonitis and peri-bronchovascular consolidations (13, 14). In both conditions, elevated C-reactive protein levels and hyper-ferritinemia have prognostic significance; meanwhile, macrophage activation, endothelial dysfunction, and vasculopathy have been hypothesized as pathogenic factors (15). SARS-CoV-2 infection has been proposed as a human pathogenic model for anti-MDA5 syndrome (16). Furthermore, it has been recently suggested to search for anti-MDA5 antibodies in patients with COVID-19 for prognostic purposes (17).

We observed a case of anti-MDA5 syndrome becoming evident after COVID-19 and then focused on reports of inflammatory myositis occurring in association with SARS-CoV-2 infections *via* a systematic literature review. Ultimately, we aim to hypothesize potential immune pathogenic mechanisms for both conditions.

## Anti-MDA5 Syndrome Following Mild COVID-19: Case Report

**Figure 1:**
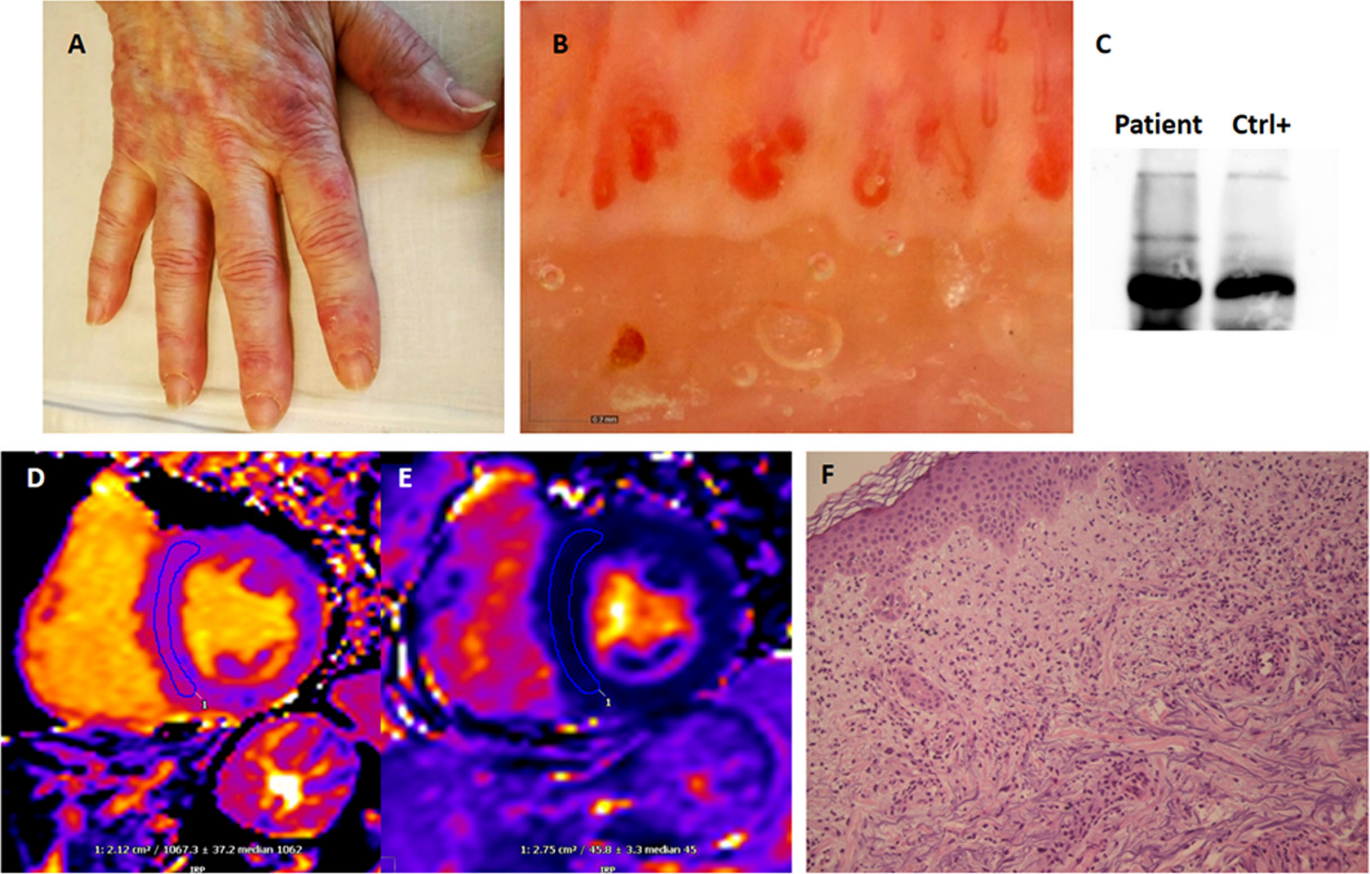
Manifestations of COVID-19-associated anti-MDA5 dermatomyositis. **(A)** Cutaneous manifestations presented as violaceous, maculo-papular lesions on both dorsal and volar sides. **(B)** Nailfold video-capillaroscopy illustrated reduced capillary density, neo-angiogenesis, and tortuous, ectasic and giant capillaries. **(C)** Anti-MDA5 antibodies detected by immunoprecipitation. **(D, E)** T1-weighted cardiac magnetic resonance images presenting increased signal intensity [native T1 = 1067 ± 37msec (NV < 1015)], which, in association with ECV = 30 ± 4% (NV < 29) and normal T2 intensity [native T2 = 46 ± 3 msec (NV < 50 msec)], indicates interstitial myocardial fibrosis and is consistent with previous myocarditis. **(F)** Skin punch biopsy of a Gottron-like lesion on the left hand showing patchy mixed superficial inflammatory infiltrated with leukocytoclastic vasculitis features (Hematoxylin and eosin, 20x magnification).

In November 2020, a 70-year-old Caucasian woman developed fever, cough, and anosmia. Four months before, she had developed an unexplained skin rash which resolved with glucocorticoids and antihistamines. She had a history of moderate-severity chronic obstructive pulmonary disease (COPD) associated with active smoking. At the time, the woman did not undergo any nasopharyngeal swab, and her symptoms resolved spontaneously in two weeks; meanwhile, her close contacts were diagnosed with COVID-19. One month after the resolution of her flu-like symptoms, she developed arthralgias and skin lesions on her face, chest, and hands; the patient denied any muscle-related symptom or weakness. She sought dermatological evaluation, and a topical treatment was started without any improvement. A SARS-CoV-2 nasopharyngeal swab proved negative and a serum test for immunoglobulin G (IgG) against SARS-CoV-2 spike protein resulted positive (96.1 AU/mL – positive if titer > 15); since the patient was not vaccinated at the time, this result demonstrates previous SARS-CoV-2 infection. For the persistence of skin rash, Gottron-like lesions on her hands (**Figure 1**), and arthralgias, she was admitted to our Department of Rheumatology and Clinical Immunology in March 2021. Blood tests proved normal except for C-reactive protein (2.1 mg/dL – normal value NV < 0.5 mg/dL), aspartate (84 IU/L – NV 5-35 IU/L) and alanine aminotransferase (133 IU/L – NV 5-35 IU/L), polyclonal hyper-gammaglobulinemia (23 g/L – 26.2%), elevated ferritin (595 ng/mL – NV 11-306 ng/mL), troponin I (19 ng/L – NV < 14 ng/L), and type B natriuretic peptide (BNP) (251 pg/mL – NV 5-100 pg/mL); creatine kinase (CK) values were normal (40 IU/L – NV < 135 IU/L), as were complement levels. Serum tests demonstrated previous hepatitis B infection with positive HBs antibody, and IgG antibodies for SARS-CoV-2 spike protein were still positive (96 AU/mL – NV < 15 AU/mL). The patient was tested for serum autoantibodies, including the myositis-associated panel: rheumatoid factor (RF), antinuclear antibodies (ANA), anti-extractable nuclear antigen (ENA) screening, anti-citrullinated protein antibodies (ACPA), antiphospholipid antibodies, and antineutrophil cytoplasm antibodies (ANCA) were negative; anti-MDA5 antibodies proved positive at both immunoblotting and immunoprecipitation analysis. A nailfold video-capillaroscopy showed reduced capillary density, neo-angiogenesis, and giant capillaries (**Figure 1**). She underwent chest CT scan revealing pulmonary emphysema, compatible with her COPD history, but not ILD. Pulmonary function tests demonstrated moderate obstruction and a severe reduction in diffusing capacity for carbon monoxide (DLCO): FVC 1.83 L – 82% of predicted, FEV1 1.12 L – 64% of predicted, and DLCO 37% of predicted. No abnormalities were found on EKG and echocardiography, but cardiac magnetic resonance imaging (MRI) findings were consistent with interstitial fibrosis as mirrored by increased native T1 mapping time (**Figure 1** **)**. A diagnosis of anti-MDA5 syndrome with cutaneous and cardiac involvement was made. Screening for malignancy was performed by chest CT scan, neck and abdominal ultrasound, urinary and cervical cytology, fecal occult blood test and resulted negative. Low-dose methylprednisolone (0.25 mg/Kg/die) and azathioprine 100 mg daily were started. Methotrexate was not prescribed due to concomitant non-alcoholic steatohepatitis and according to the patient preferences and disability. After six months, skin lesions improved but did not completely resolve, with a persistent tenuous heliotrope rash and V-neck sign. Furthermore, active myocarditis was retrieved at cardiac MRI, as demonstrated by mild increase in native T2 mapping time. Azathioprine was stopped, and mofetil mycophenolate mofetil 3 grams daily was started (18).

## Study Search Strategy and Selection

The Medline database was accessed from PubMed and systematically searched for articles published in English between January 1st, 2020 and March 31st, 2022. We followed the search strategy and article selection process illustrated in the flowchart in **Supplementary Figure 1**, according to the recommendations of the PRISMA statement (19). Only peer-reviewed articles in English accepted for publication that included case reports and case series were included in this search. Two reviewers (FM and AT) evaluated all potentially relevant articles independently and summarized them. They discussed any area of uncertainty, screened the full text reports, and decided whether these met the inclusion criteria while resolving any disagreement through discussions. Neither of the authors were blind to the journal titles or to the study authors or institutions.

## Results

Eleven cases of inflammatory myositis temporally related to COVID-19 have been reported (20–27); the main features are illustrated in **Table 1**. In nine patients, new-onset inflammatory myositis followed SARS-CoV-2 infection; two cases of disease relapse following COVID-19 have been reported: one at pediatric age (22) (in a child who experienced a skin-only relapse of juvenile DM) and one in the adult setting (20) (in a patient positive for both anti-Mi-2 and anti-PM/Scl antibodies). Three out of eleven cases were juvenile DM (22, 26), in the other 8 adult cases the median age was 58 (interquartile range – IQR 50-64) years; a strong female predominance was observed (8/11 cases). In 9/11 reports, the severity of COVID-19 is described: 3/9 patients developed overt pneumonia, one of them succumbing to the infection (20); a flu-like illness was experienced in 4/9 cases, whereas two patients (22, 26) were completely asymptomatic. Apart from the case described by Gokhale (20) et al., none of the patients required specific therapies, oxygen supplementation nor mechanical ventilation for COVID-19. Time from the diagnosis of SARS-CoV-2 infection to the onset of inflammatory myositis was less than two months, and in four patients (20, 24, 26) rheumatologic manifestations were concomitant with the diagnosis of the infection. DM-related skin involvement was present in all the patients, being the only manifestation in one case of relapsed juvenile DM (22). Myopathy was noticed in 10/11 cases, generally in the form of a mild/moderate proximal muscle weakness; cardiomyopathy was only detected in one patient with systemic lupus erythematosus-dermatomyositis overlap (21). Pulmonary involvement was present or suspected (based on worsening dyspnea) in five cases (20, 21, 23, 24). There was no report of cancer-related DM.

**Table 1:** Relevant clinical features of previously reported cases of DM following SARS-CoV-2 infection.

Reference	Patient’s characteristics: sex, age, race	Past medical history	Medications	SARS-CoV-2 information (time and severity)	Therapy for COVID-19	Clinical manifestations of DM	Autoimmunity and diagnosis	Therapy for DM	Outcome
Shahidi Dadras, 2021, Clin Case Rep. (21)	F, 58 Asian	Diabetes mellitus Hypothyroidism Coronary artery disease	Losartan Aspirin Metformin Levothyroxine Doxepin	45 days before Flu-like	Home supportive care	Constitutional symptoms Skin manifestations Fever Muscle weakness Pneumonia Cardiomyopathy	Seronegative DM-SLE overlap	PDN 60 mg/d MTX 15 mg/wk HCQ 400 mg/d	Improved
Liquidano-Perez, 2021, Pediatr Neurol. (26)	F, 4 Latino	Negative	N.A.	Concomitant Asymptomatic	N.A.	Muscle weakness Skin manifestations Dyspnea (myogenic)? Esophageal dysmotility	ANA 1:320 Anti-RNP/Sm Anti-Scl70 Anti-Sm Juvenile DM	IVIG 1 g/Kg MPN 0.7 mg/Kg/d, then pulses HCQ MTX CsA	Respiratory deterioration requiring mechanical ventilation. Gradual improvement
Rodero, 2022, J Clin Immunol (22)	F, 15 Unknown	Negative	N.A.	Two weeks before Unknown	No	Constitutional symptoms Arthritis Muscle weakness Skin manifestations and telangiectasias	Seronegative Juvenile DM	IVIG Steroid TOFA 5 mg bid	Remission
Rodero, 2022, J Clin Immunol (22)	F, 12 Unknown	DM, diagnosed eight years before	N.A.	Two weeks before Asymptomatic	No	*8 years before*: Muscle weakness Skin manifestations *Relapse*: Skin manifestations	Seronegative Juvenile DM	First episode: Steroids MTX Relapse: IVIG Steroids	Remission
Keshtkarjahromi, 2021, BMC Rheumatol (23)	F, 65 Caucasian	Psoriasis Hypertension Hyperlipidemia	Amlodipine Atorvastatin Buspirone Pantoprazole	Two months before Unknown	N.A.	Constitutional symptoms Muscle weakness Skin manifestations Dyspnea Arthralgia MAS	ANA Anti-MDA5 Anti-Ro52 DM	PDN 60 mg/d For MAS: MPN boluses, IVIG and mechanical ventilation	Died due to MAS
Gokhale, 2020, J Assoc Physicians India (20)	M, 64 Asian	N.A.	N.A.	Concomitant Pneumonia	IV antibiotic HCQ Ivermectin	Muscle weakness Skin manifestations Fever	ANA 1:320 homogeneous DM	IVIG 2 g/Kg PDN 1 mg/Kg/d MMF 1.5 g/d	Improved
Gokhale, 2020, J Assoc Physicians India (20)	F, 50 Asian	N.A.	N.A.	Concomitant Pneumonia	N.A.	Muscle weakness Skin manifestations OP (COVID-19)? Fever	Anti-MDA5 Anti-SAE-1 DM	MPN CYC 1 g MTX 15 mg/wk	Died due to COVID-19
Gokhale, 2020, J Assoc Physicians India (20)	M, 50 Asian	DM, diagnosed eight years before	PDN 5 mg qd AZA 50 mg bid	Convalescent (positive IgM and IgG antibodies towards SARS-CoV-2 but negative swab) Pneumonia	N.A.	8 years before: Skin manifestations Muscle weakness Relapse: Muscle weakness Skin manifestations Pneumonia (COVID-19)?	Anti-Mi-2 Anti-PM/Scl DM	MTX PDN IVIG	Improved
Borges, 2021, Rheumatology (Oxford) (25)	F, 36 Latino	N.A.	N.A.	Two weeks before Flu-like	N.A.	Skin manifestations Muscle weakness Raynaud’s phenomenon	ANA 1:640, fine speckled Anti-Mi-2 DM	MPN	Improved
Okada, 2021, Rheumatology (Oxford) (27)	F, 64 Asian	N.A.	N.A.	One month before Fever	No	Skin manifestations Muscle weakness	Anti-NXP2 DM	MPN 1 g, then oral PDN 60 mg/d AZA 50 mg/d	Improved
Ho, 2021, JAAD Case Rep. (24)	M, 58 Hispanic	Negative	No	Concomitant Flu-like	Supportive care	Constitutional symptoms Muscle weakness Pneumonitis Skin manifestations Pulmonary embolism	Negative serology Biopsy-proven DM	MPN pulses Oral PDN MTX 10 mg/wk	Improved

ANA, antinuclear antibodies; AZA, azathioprine; bid, two times per day; CsA, cyclosporin A; CYC, cyclophosphamide; d, day; DM, dermatomyositis; F, female; HCQ, hydroxychloroquine; IV, intravenous; IVIG, intravenous immunoglobulins; MAS, macrophage activation syndrome; MMF, mofetil mycophenolate; MPN, methylprednisolone; MTX, methotrexate; N.A., not applicable or unknown; OP, organizing pneumonia; PDN, prednisolone; qd, once per day; SLE, systemic lupus erythematosus; TOFA, tofacitinib; wk, week.

In terms of autoimmune profiling, 4/11 cases (21, 22, 24) tested negative for serum autoantibodies, including two pediatric cases (22) and a lupus/dermatomyositis overlap syndrome (21). Two patients (20, 23) tested positive for anti-MDA5 antibodies: one of them (also positive for anti-Ro52 antibodies) underwent fatal macrophage activation syndrome (23), the other (also positive for anti-SAE-1 antibodies) died due to pneumonia (20). Two patients were positive for anti-Mi-2 antibodies: one of them was diagnosed new-onset DM (25), the other (also positive for anti-PM/Scl antibodies) experienced disease relapse (20). Moreover, one case of anti-NXP2 DM was described (27). Eight out of eleven patients experienced favorable outcome regarding both inflammatory myositis and COVID-19. One case of juvenile DM required mechanical ventilation due to respiratory deterioration, but gradual improvement followed thereafter (26). As already stated, two deaths were observed: one following macrophage activation syndrome (23), the other attributable to severe SARS-CoV-2 pneumonia (20); both patients had tested positive for anti-MDA5 autoantibodies.

## Discussion

### SARS-CoV-2 at the Crossroads of Anti-Viral Immunity and Autoimmunity: The Role of MDA5

Viral infections have been hypothesized as environmental triggers in the pathogenesis of autoimmune diseases, including inflammatory myopathies (28), likely through molecular mimicry (1). Three immunogenic linear epitopes with high sequence identity to SARS-CoV-2 proteins have been recently recognized in patients with DM (29) and may explain how SARS-CoV-2 could trigger an autoimmune response in predisposed subjects, leading to autoantibody production. Further, autoantibodies directed towards specific antiviral signaling proteins (i.e., MDA5 and RIG-I) have been recently described by our group in a cohort of patients affected by COVID-19 (28). Notably, SARS-CoV-2 replication occurs through the synthesis of double-stranded RNA intermediates (dsRNA). RIG-I and MDA5 are pattern recognition receptors involved in innate antiviral immunity, and act as major sensors for dsRNA intermediates. While MDA5 recognizes long dsRNAs, RIG-I binds to shorter dsRNA fragments (30, 31). After binding to the viral dsRNA, MDA5/RIG-I activate a Janus kinase (JAK)/Signal Transducer and Activator of Transcription (STAT) signaling pathway leading to type I IFN production, which is an essential cytokine in antiviral immune response (32). RNA viruses, as well as IFN itself, can upregulate the expression of MDA5 in infected cells, thus amplifying the whole process. After viral-induced cell lysis, dsRNA-MDA5/RIG-I complexes are released in the extracellular space, where they act as cryptic antigens, leading to autoantibody production (14). Through the generation of ‘new self’ epitopes, SARS-CoV-2 could elicit the synthesis of autoantibodies against antiviral signaling proteins (28). Moreover, apart from inducing type I IFN synthesis, RIG-I seems also to inhibit viral replication in an IFN-independent manner (33). It is unknown whether anti-MDA5 and anti-RIG-I antibodies possess any pathogenic effect, or they just represent the epiphenomenon of an aberrant activation of the immune response.

### Type I IFN in the Pathogenesis of COVID-19

The severity of COVID-19 has been reported to be inversely related to the IFN production: if compared with mild-moderate forms, severe and life-threatening infections display an impaired type I IFN activity, a reduced viral clearance, and a delayed hyper-inflammatory response (34). Inborn errors of the type I IFN pathway have been associated with more severe forms of COVID-19, and severe SARS-CoV-2 infection led to new diagnosis of such type of immune deficiencies in a study cohort (35). Moreover, SARS-CoV-2 is able to evade the immune defense by antagonizing various steps involved in type I IFN synthesis (36, 37), including the direct inhibition of RIG-I and MDA5 by specific viral proteins (38). Studies conducted on animal models found that early IFN production would be crucial in producing an effective and protective antiviral response towards SARS-CoV-2, leading to the subsequent development of mild forms of COVID-19 (39). Autoantibodies directed towards type I IFN have been described in patients with severe SARS-CoV-2 pneumonia; by neutralizing IFN molecules, these antibodies would tip the balance in favor of the virus, thereby reducing viral clearance (40). According to these data, low type I IFN activity is associated with more severe COVID-19; vice versa, we could hypothesize that a strong type I IFN signature might be beneficial in orchestrating the antiviral immune response, leading to milder forms of disease.

### Type I IFN in the Pathogenesis of Autoimmunity and the Anti-MDA5 Syndrome

Beyond their crucial role in viral clearance, type I IFN levels are elevated in sera of patients with autoimmune diseases, including systemic lupus erythematosus, rheumatoid arthritis, and DM (41, 42). Treatment with IFN-alpha (e.g. for HCV hepatitis or multiple sclerosis) has been associated with the appearance of serum autoantibodies, such as antinuclear antibodies and anti-dsDNA (43). Moreover, cases of new-onset inflammatory myositis following IFN-alpha administration have been described (44). It is worth mentioning that type I IFN levels correlate with disease activity in patients with DM and their downregulation can predict response to therapy (45). Finally, a high type I IFN signature might have a pathogenic role in vasculopathy and interstitial lung damage, which are prominent features of DM (46). If compared with other inflammatory myopathies, anti-MDA5 syndrome shows an even stronger type I IFN signature (46–48). Increased levels of MDA5 expression are found in peripheral mononucleated blood cells of patients with anti-MDA5 syndrome (47), and a possible role for type I IFN in the pathogenesis of the disease has been recently proposed (48). Furthermore, a peculiar population of autoreactive B cells has been recently described in a cohort of patients with severe anti-MDA5 syndrome. These lymphocytes synthetize monoclonal autoantibodies that can stimulate IFN-gamma (a type II IFN) production in peripheral blood mononucleated cells; notably, these antibodies are not directed towards MDA5 (49). The interplay between type I and II IFNs in both antiviral response and autoimmune pathogenesis is currently under investigation. Little is known about the relationship between MDA5 activation, type I IFN signature, autoantibody synthesis, and the immune pathogenic mechanisms that lead to the development of anti-MDA5 syndrome. Moreover, since autoimmune complications have been found in only a minority of patients affected by COVID-19, predisposing factors remain largely unknown.

### Case Discussion and Pathogenic Hypothesis

We have recently described autoantibodies directed towards antiviral signaling proteins (e.g., MDA5 and RIG-I) in a cohort of patients affected by mild COVID-19; however, none of the aforementioned patients developed any feature suggestive of systemic autoimmune disease (28).

Through the recognition of viral dsRNA and the activation of a type I IFN response, MDA5 could link SARS-CoV-2 infection and subsequent anti-MDA5 syndrome (50). In our case, overt anti-MDA5 syndrome was preceded by a mild (flu-like) form of SARS-CoV-2 infection. A type I IFN-driven response may have exerted an effective antiviral activity, explaining the mild course of COVID-19 reported by our patient. We may hypothesize that the woman went through a prominent type I IFN response, with subsequent enhanced viral clearance. Besides having antiviral activity, this strong cytokine signature could have triggered the immune mechanisms underlying anti-MDA5 syndrome, which is an IFN-mediated disease (47, 48). Furthermore, COVID-19 could have revealed a pre-existing autoimmune condition/predisposition: the woman might have had previously DM-like skin rash, and SARS-CoV-2 infection could have unveiled overt disease by eliciting a strong IFN signature. There is uncertainty in the patient’s past medical history, and no laboratory tests were performed before referral to our clinic: this represents a limitation for any possible conclusion, we must be aware that most cases will remain with some blind spots that need to be recognized.

High type I IFN levels are associated with an increased risk and are predictors of disease activity in some IFN-mediated autoimmune diseases, such is the case of anti-MDA5 syndrome (45, 47). A strong IFN signature might be related to an enhanced viral clearance and, consequently, to a mild course of SARS-CoV-2 infection. In this view, our patient would be paradigmatic, with mild COVID-19 preceding DM onset/flare. Thus, autoimmunity resulting from an exuberant and dysregulated activation of the immune system could harbor parallel protective effects, due to an increased immune surveillance against viral infections. Notwithstanding, we must be aware that this interpretation is probably simplistic. The presence of anti-MDA5 antibodies has been recently correlated with a severe course of SARS-CoV-2 infection in a Chinese cohort, and it has also been proposed that autoantibody production might occur after the release of the antigen from infected cells (17). As reported in our systematic review, two out of two patients positive for anti-MDA5 antibodies died. In one case (23) anti-Ro52 antibodies, which are associated with rapidly progressive ILD and worse prognosis (51), were also present; the woman succumbed to macrophage activation syndrome, and there’s no reason to attribute her death to COVID-19, since anti-MDA5 syndrome appeared two months after recovery from the infection. Meanwhile, the patient described by Gokhale et al. (20) tested positive for anti-SAE1 antibodies, which are associated with ILD and increased cancer risk (52). It is unclear whether the woman died due to SARS-CoV-2 pneumonia or to anti-MDA5 syndrome-associated rapidly progressive-ILD, since both conditions can manifest with organizing pneumonia features at chest CT scan (13). Furthermore, a more severe phenotype of anti-MDA5 syndrome has been described in Asian ancestry (53), such is the case of this patient. Taken together, these demographic, clinical, and serologic differences could partially explain the diversities between previously described cases and our report of anti-MDA5 syndrome following mild SARS-CoV-2 infection.

Similarities between COVID-19 and anti-MDA5 syndrome might implicate changes in treatment strategies in both conditions (16). Further research is required to elucidate the mechanisms that lead to anti-MDA5 autoantibodies synthesis in a subset of SARS-CoV-2 infected patients. Also, there is an urge to understand which predisposing factors (e.g., genetically determined) couple autoantibody production with the development of anti-MDA5 syndrome in only a minority of cases. Furthermore, little is known about if (and how) anti-MDA5 antibodies might influence the outcome of COVID-19. Finally, the balance between immune activation vs. immune tolerance is still extensively unexplored.

## Conclusions

Type I IFN is an essential component of the immune response towards viral infections, and a strong IFN signature has been described in various connective tissue diseases, including anti-MDA5 syndrome. Apart from triggering the synthesis of autoantibodies, SARS-CoV-2 might be able to elicit an autoimmune response involved in inflammatory myositis pathogenesis, associated to the type I IFN-rich molecular *milieu* promoted by the virus itself. Both *de novo* anti-MDA5 syndrome development and disease relapses could occur through such immune mechanisms. Ultimately, clinicians should be aware that autoimmune phenomena, ranging from isolated autoantibody positivity to overt systemic rheumatologic syndromes, are a possible complication of even mild or asymptomatic SARS-CoV-2 infections, and that the dogmatic separation between infections and chronic inflammation should be dynamically reconsidered.

## Data Availability Statement

The original contributions presented in the study are included in the article/**Supplementary Material**. Further inquiries can be directed to the corresponding author.

## Ethics Statement

Written informed consent was obtained from the individual(s) for the publication of any potentially identifiable images or data included in this article.

## Author Contributions

All authors equally contributed to the submitted version of the work, by data achieving, drafting or revising the manuscript.

## Conflict of Interest

The authors declare that the research was conducted in the absence of any commercial or financial relationships that could be construed as a potential conflict of interest.

## Publisher’s Note

All claims expressed in this article are solely those of the authors and do not necessarily represent those of their affiliated organizations, or those of the publisher, the editors and the reviewers. Any product that may be evaluated in this article, or claim that may be made by its manufacturer, is not guaranteed or endorsed by the publisher.
