# Supplementary material for: Anti-MDA5 Antibody Linking COVID-19, Type I Interferon, and Autoimmunity: A Case Report and Systematic Literature Review
Source: Front Immunol. 2022 Jun 27;13:937667. doi: 10.3389/fimmu.2022.937667 (PMC9271786; doi:10.3389/fimmu.2022.937667)
Supplement: Supplementary file 1 [file DataSheet_1.docx]

**
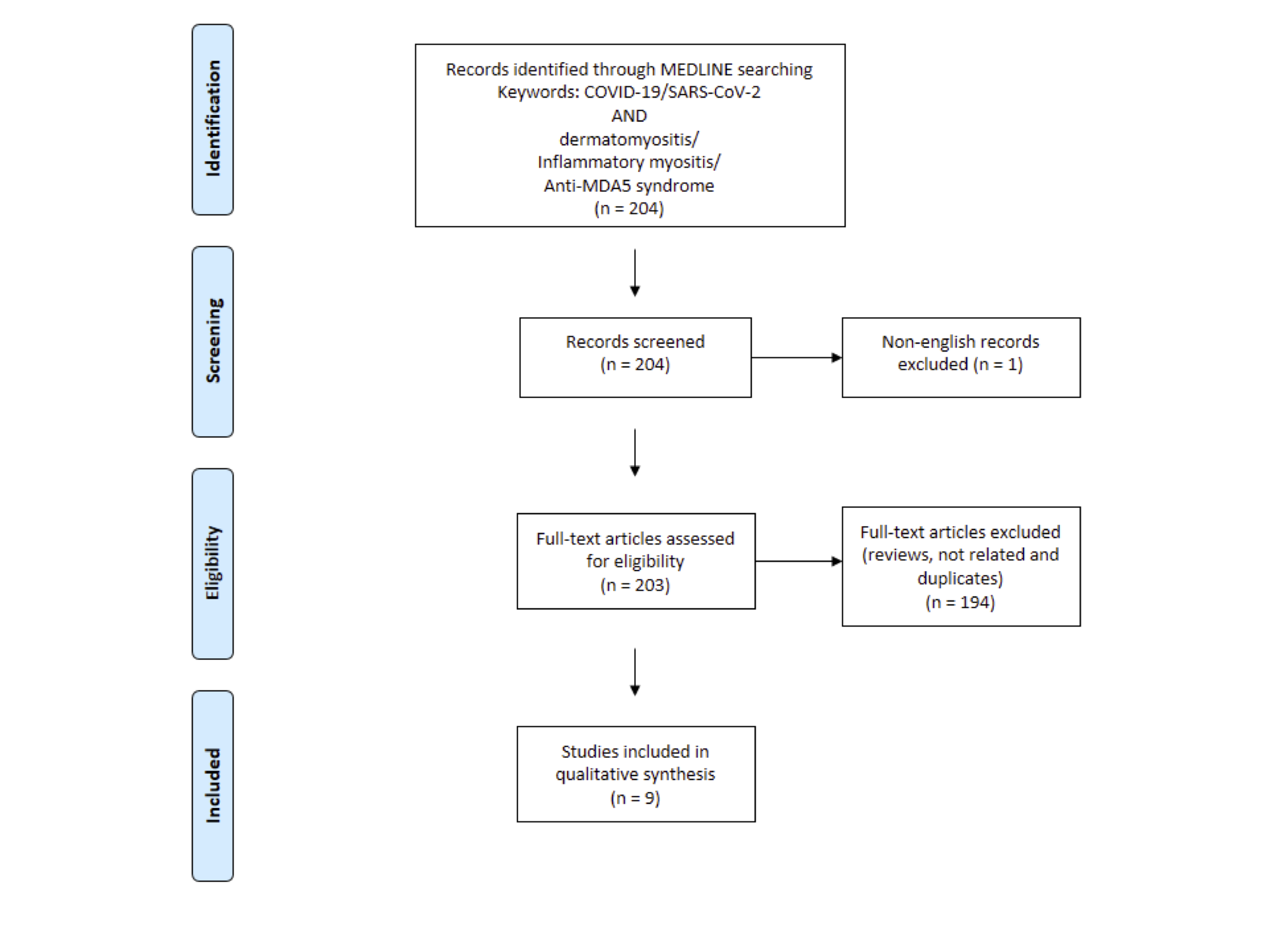
Supplementary Figure 1.** Flowchart showing the study selection process according to preferred reporting items for systematic reviews and meta-analyses (PRISMA).
